# Complete chloroplast genome of *Sphaeropteris brunoniana* (Cyatheaceae)

**DOI:** 10.1080/23802359.2020.1787893

**Published:** 2020-07-23

**Authors:** Ming Zhu, Guohua Zhao, Jingyao Ping, Yingyi Liang, Peipei Feng, Yingjuan Su, Ting Wang

**Affiliations:** a School of Life Sciences, South China Agricultural University, Guangzhou, China; b Fairylake Botanical Garden, Shenzhen & Chinese Academy of Sciences, Shenzhen, China; c School of Life Sciences, Sun Yat-sen University, Guangzhou, China; d Research Institute of Sun Yat-sen University in Shenzhen, Shenzhen, China; e Guangdong Provincial Key Laboratory of Applied Botany, South China Botanical Garden, Chinese Academy of Sciences, Guangzhou, China

**Keywords:** *Sphaeropteris brunoniana*, chloroplast genome, phylogeny

## Abstract

Illumina sequencing was employed to determine the complete chloroplast (cp) genome sequence of *Sphaeropteris brunoniana* (*S. brunoniana*), which is a relict fern. The cp genome of *S. brunoniana* is indeed a circular DNA molecule with 156,659 bp. It includes an inverted repeats (IRs) pair with 24,011 bp each and two single-copy regions with 86,196 bp and 22,441 bp, respectively. Additionally, the genome contains 117 unique genes encoding 85 proteins, 28 tRNAs, four rRNAs. Pseudogenes of ycf66 and trnT-UGU are also detected in this genome.Bayesian phylogenetic tree strongly supports the deduction that *S. brunoniana* belongs to Cyatheaceae. To date, this is the first cp genome for the genus *S. brunoniana.*

*Sphaeropteris brunoniana* (Wallich ex Hooker) R. M. Tryon is essentially a tree fern that belongs to genus *Sphaeropteris* (Cyatheaceae). Its trunk height is typically 10–20 m (Zhang and Nishida 2013). Fronds can bebi- or tripinnate and their lengths can be 2–3 m. The rachis and stipe are smooth or finely warty and they tend to exhibit pale brown or brown basal scales. Also, rachis and stipe are usually thin and have setiferous edges. Soriis usually observed near the midveins of fertile pinnules and often fill the lower lamina; indusia are absent (Large and Braggins 2004). *Sphaeropteris brunoniana* is distributed in northeastern part of India, Bangladesh, Burma, and Vietnam. Meanwhile, China is the northern limit of its distribution. Specifically, a total of 14 species and two varieties have been observed in China, and *S. brunoniana* is currently the only Sphaeropteris species observed in mainland China (Zhang 2004). *Sphaeropteris brunoniana* plays a key role in classification of Cyatheaceae, as well as its phylogeny (Ching 1978; Wang et al. 2003). Identification of its complete cp genome sequence is of great significance to the chloroplast (cp) phylogenomics of Cyatheaceae and protection of this only Sphaeropteris species in mainland China.

Fresh leaves of *S. brunoniana* were sampled from the living collection at Fairylake Botanical Garden (22°34′43.10″N, 114°09′55.98″E). The specimen is stored in Herbarium of South China Agricultural University (SCAUB; voucher: M Zhu 201910). The Tiangen Plant Genome DNA Kit (Tiangen Biotech Co., Ltd., Beijing, China) was utilized to extract DNA. Sequencing was carried out on a HiSeq2500 (Illumina Inc., San Diego, CA), which generated a total of 11,640,160 raw reads. After trimming to remove low quality reads, we employed Velvet (Zerbino and Birney 2008) to assemble the remaining reads into contigs. The resulting contigs were then aligned against the cp genome of *Alsophila spinulosa* (NC_012818). PCR amplification was utilized to fill any remaining gaps (Liu et al. 2018). (Liu et al., 2018) Annotations were performed using DOGMA (Wyman et al. 2004) and tRNAscan-SE programs (Lowe and Eddy 1997). All annotated cp genomes were rechecked using Geneious Prime (Biomatters, Auckland, New Zealand) based on previously published cp genome *Alsophila spinulosa* (NC_012818). We selected 15 cp genomes to reconstruct the phylogenetic tree of 15 species of fern. The program MAFFT plugin in PhyloSuite v1.2.1 (Zhang et al. 2020) was used to create a multiple sequence alignment of the complete cp genome of *S. brunoniana* with those of other 14 plants downloaded from GenBank. The best-fit model is GTR + F+I + G4 chosen according to BIC, and the phylogenetic tree was constructed using MrBayes method by PhyloSuite (Figure 1). *Mankyua chejuensis* (NC_017006) was selected as outgroup.

**Figure 1.**
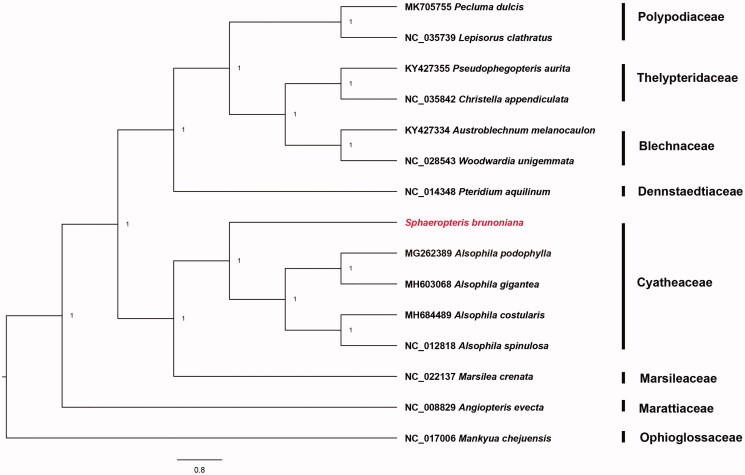
Bayesian phylogenetic tree of 15 fern species. *Mankyua chejuensis* was selected as outgroup.

The complete cp genome of *S. brunoniana* is essentially a circular DNA molecule with 156,659 bp (GenBank accession number: MT543220). It exhibits a quadripartite structure with two single copy (LSC with 86,196 bp and SSC with 22,441 bp) regions, which are separated by a pair of inverted repeats (IRs, 24,011 bp each). The cpDNA involves 133 genes, among which 89 are protein-coding ones, eight are rRNA ones, 33 are tRNA ones, and three are pseudogenes. In the cpDNA, 117 are unique and others are completely duplicated in IRs. Pseudogenes of ycf66 and trnT-UGU are alsodetected in this genome. Additionally, 12 genes involve one intron only, while three genes (*ycf3*, *clpP*, and *rps12*) contain two. The GC contents in LSC, SSC, and IR regions were 39.2%, 43.2%, and 38.1%, respectively. Meanwhile, the overall GC content of the cp genome was 40.3%. Phylogenetic analysis strongly supports the deduction that *S. brunoniana* belongs to Cyatheaceae (Figure 1). The cp genome of *S. brunoniana* is of great significance to phylogenetic studies and cp genomics of ferns.

## Data Availability

The data that support the findings of this study are available in NCBI at https://www.ncbi.nlm.nih.gov/. These data were derived from the following resources available in the public domain: https://www.ncbi.nlm.nih.gov/nuccore/KY427334, NC_028543, MH684489, MH603068, MT543220, MG262389, NC_012818, NC_014348, NC_008829, NC_022137, NC_017006, NC_035739, MK705755, NC_035842, KY427355.
